# Increased seawater temperature increases the abundance and alters the structure of natural *Vibrio* populations associated with the coral *Pocillopora damicornis*

**DOI:** 10.3389/fmicb.2015.00432

**Published:** 2015-05-18

**Authors:** Jessica Tout, Nachshon Siboni, Lauren F. Messer, Melissa Garren, Roman Stocker, Nicole S. Webster, Peter J. Ralph, Justin R. Seymour

**Affiliations:** ^1^Plant Functional Biology and Climate Change Cluster, University of TechnologySydney, NSW, Australia; ^2^Ralph M. Parsons Laboratory, Department of Civil and Environmental Engineering, Massachusetts Institute of TechnologyCambridge, MA, USA; ^3^Australian Institute of Marine ScienceTownsville, QLD, Australia

**Keywords:** *Vibrio*, *Vibrio coralliilyticus*, *Pocillopora damicornis*, corals, heat stress, pathogen

## Abstract

Rising seawater temperature associated with global climate change is a significant threat to coral health and is linked to increasing coral disease and pathogen-related bleaching events. We performed heat stress experiments with the coral *Pocillopora damicornis*, where temperature was increased to 31°C, consistent with the 2–3°C predicted increase in summer sea surface maxima. 16S rRNA amplicon sequencing revealed a large shift in the composition of the bacterial community at 31°C, with a notable increase in *Vibrio*, including known coral pathogens. To investigate the dynamics of the naturally occurring *Vibrio* community, we performed quantitative PCR targeting (i) the whole *Vibrio* community and (ii) the coral pathogen *Vibrio coralliilyticus*. At 31°C, *Vibrio* abundance increased by 2–3 orders of magnitude and *V. coralliilyticus* abundance increased by four orders of magnitude. Using a *Vibrio*-specific amplicon sequencing assay, we further demonstrated that the community composition shifted dramatically as a consequence of heat stress, with significant increases in the relative abundance of known coral pathogens. Our findings provide quantitative evidence that the abundance of potential coral pathogens increases within natural communities of coral-associated microbes as a consequence of rising seawater temperature and highlight the potential negative impacts of anthropogenic climate change on coral reef ecosystems.

## Introduction

The health and function of coral reefs is profoundly influenced by microorganisms, which often form species-specific associations with corals (Rohwer et al., 2002; Rosenberg et al., 2007; Mouchka et al., 2010). These ecological relationships can be mutualistic, commensal or pathogenic (Rosenberg et al., 2007), and diseases caused by pathogenic microbes have been identified as a key threat to coral reefs globally (Bourne et al., 2009; Burge et al., 2014). Diseases including white syndrome – which causes bleaching and lysis (Kushmaro et al., 1996; Ben-Haim et al., 2003a; Rosenberg and Falkovitz, 2004), white band (Ritchie and Smith, 1998; Aronson and Precht, 2001), white plague (Thompson et al., 2001), white pox (Patterson et al., 2002), black band (Frias-Lopez et al., 2002; Sato et al., 2009), and yellow band (Cervino et al., 2008) have all been attributed to microorganisms and have led to mass mortalities and significant loss of coral cover (Bourne et al., 2009).

There is evidence that the occurrence and severity of coral disease outbreaks is increasing globally (Harvell et al., 2004; Bruno et al., 2007; Mydlarz et al., 2010), potentially due to environmental stressors associated with phenomena such as increases in seawater temperature (Mouchka et al., 2010; Ruiz-Morenol et al., 2012). Heat stress may compromise the health of corals, leading to enhanced susceptibility to disease (Hoegh-Guldberg, 1999; Hoegh-Guldberg and Hoegh-Guldberg, 2004; Jokiel and Brown, 2004), or increase the abundance and/or virulence of pathogens (Vega Thurber et al., 2009; Vezzulli et al., 2010; Kimes et al., 2011). Increases in seawater temperature have been shown to change the composition and functional capacity of coral-associated microbial communities, including shifts to an elevated state of virulence, and pathogenicity (Vega Thurber et al., 2009).

While diverse groups of microbes, including bacteria, fungi, and viruses have been implicated in several coral diseases, one bacterial genus in particular has become a recurrent feature within coral disease research. *Vibrio* are globally distributed marine *Gammaproteobacteria* (Pollock et al., 2010), which harbor a diverse virulence repertoire that enables them to be efficient and widespread pathogens of a wide range of marine species (Santos Ede et al., 2011), including shell-fish (Jeffries, 1982), fish (Austin et al., 2005), algae (Ben-Haim et al., 2003b), mammals (Kaper et al., 1995; Shapiro et al., 1998; Oliver, 2005), and corals (Ben-Haim et al., 2003b). White syndrome in *Montipora* corals is caused by *V. owensii* (Ushijima et al., 2012), white band disease II in *Acropora cervicornis* has been attributed to *V. charchariae* (synonym for *V. harveyi*; Gil-Agudelo et al., 2006; Sweet et al., 2014), and a consortium of *Vibrio* are responsible for yellow band disease (Cervino et al., 2008; Ushijima et al., 2012). Furthermore, *V. shiloi* and *V. coralliilyticus* are the causative agents of bleaching in the coral species *Oculina patagonica* (Kushmaro et al., 1996, 1997, 1998; Toren et al., 1998) and the cauliflower coral *Pocillopora damicornis* (Ben-Haim and Rosenberg, 2002; Ben-Haim et al., 2003b), respectively.

Laboratory experiments using cultured isolates of *V. shiloi* (Kushmaro et al., 1996, 1997) and *V. coralliilyticus* (Ben-Haim and Rosenberg, 2002; Ben-Haim et al., 2003b) have fulfilled Koch’s postulates, with each species proven to be the causative agent of coral bleaching. *V. shiloi* causes bleaching in *O. patagonica* by using chemotaxis toward the coral mucus, before adhering to the coral surface and penetrating the epidermis (Banin et al., 2001). After colonization of the coral, cell multiplication occurs followed by production of the Toxin P molecule, which inhibits photosynthesis in the symbiotic zooxanthellae, resulting in coral bleaching, and tissue loss (Rosenberg and Falkovitz, 2004). Similarly, *V. coralliilyticus* causes bleaching, lysis and tissue loss in the coral *P. damicornis* (Ben-Haim and Rosenberg, 2002; Ben-Haim et al., 2003b; Meron et al., 2009; Garren et al., 2014). The mechanism behind *V. coralliilyticus* infection also includes motility and chemotaxis (Ben-Haim and Rosenberg, 2002; Ben-Haim et al., 2003b) and involves the post-colonization production of a potent extracellular metalloproteinase, which causes coral tissue damage (Ben-Haim and Rosenberg, 2002; Ben-Haim et al., 2003b). Another key similarity in the infection and bleaching mechanisms of *V. shiloi* and *V. coralliilyticus* is an increased infection rate under elevated seawater temperatures (Toren et al., 1998; Ben-Haim and Rosenberg, 2002; Ben-Haim et al., 2003b).

Heat stress can enhance coral disease by increasing host susceptibility to infection by pathogens (Bourne et al., 2009; Mouchka et al., 2010) or altering the behavior and virulence of pathogenic bacteria (Kushmaro et al., 1998; Banin et al., 2001; Ben-Haim and Rosenberg, 2002; Ben-Haim et al., 2003a,b; Koren and Rosenberg, 2006; Bourne et al., 2008; Kimes et al., 2011; Santos Ede et al., 2011). Notably, *V. shiloi* can only be isolated from bleached corals during summer months (Kushmaro et al., 1998) and laboratory experiments have shown that this species causes bleaching at an accelerated rate above 29°C, yet has negligible effect at 16°C (Kushmaro et al., 1998). Similarly, tissue loss caused by *V. coralliilyticus* is most rapid at elevated temperatures between 27 and 29°C (Ben-Haim and Rosenberg, 2002; Ben-Haim et al., 2003b). Seawater temperatures above 27°C have also been shown to play a direct role in the up-regulation of several *V. coralliilyticus* virulence genes, including factors involved in host degradation, secretion, antimicrobial resistance, and motility (Kimes et al., 2011). Up-regulation of motility is particularly notable as both *V. shiloi* and *V. coralliilyticus* exhibit enhanced chemotactic capacity at elevated temperatures (Banin et al., 2001; Garren et al., 2014). Heat-stressed corals also increase the production and release of signaling compounds including dimethylsulfoniopropionate (DMSP) at elevated temperature, further enhancing the ability of pathogens to locate, and colonize heat-stressed corals (Garren et al., 2014).

To date, our understanding of coral-associated *Vibrio* dynamics under elevated seawater temperatures has been solely derived from laboratory-based experiments using cultured isolates (Kushmaro et al., 1998; Toren et al., 1998; Banin et al., 2001; Ben-Haim and Rosenberg, 2002; Ben-Haim et al., 2003b; Garren et al., 2014). However, there is currently little understanding of how native communities of *Vibrio*, occurring within diverse natural assemblages of bacteria, will respond to elevated seawater temperatures. Understanding the dynamics of *Vibrio* populations within this complex, but also more realistic, scenario is important because it is very probable that Vibrios living in co-habitation with other competing and interacting species, will display different dynamics to those displayed by cultured isolates under laboratory conditions. For instance, inter-species antagonistic interactions among bacteria can strongly influence the growth and proliferation of other *Vibrio* species (Long et al., 2005), and we may expect similar ecological complexities to also occur within the coral holobiont. Here, we examined changes in the *Vibrio* population within a natural, mixed community of bacteria associated with the coral species *P. damicornis* on Heron Island, the Great Barrier Reef, Australia, and demonstrate that heat stress increases the abundance and changes the composition of potentially pathogenic *Vibrio* populations associated with corals.

## Materials and Methods

### Heat Stress Experiment

Three separate colonies (denoted A, B, and C) of the coral species *P. damicornis* were collected from within the Heron Island lagoon, on the Great Barrier Reef, Australia (23°26′41^′′^S, 151°54′47^′′^E), and translocated to the Heron Island Research Station. Colonies were placed into flow-through aquaria, which circulated water pumped from the reef flat to the Heron Island Research Station. The colonies were fragmented into 48 nubbins using bone cutters and acclimated for 8 days across six flow-through experimental tanks. The placement of the nubbins from each colony within each tank and the position of the tanks were randomized. During the experiment, three tanks were maintained at the ambient seawater temperature (22°C) experienced on the reef flat (control), while the remaining three tanks were exposed to a heat stress treatment, which involved the incremental ramping of seawater temperature by 1.5°C each day for seven consecutive days using one 25W submersible aquarium heater (Aqua One, Ingleburn, NSW, Australia) per tank, until a final temperature of 31°C was reached. Water was circulated in the tanks using one 8W maxi 102 Powerhead pump (Aqua One, Ingleburn, NSW, Australia) per tank. This temperature increase is in line with the predicted 2–3°C increases above current summer average seawater temperature (Hoegh-Guldberg, 1999, 2004; Berkelmans et al., 2004; Hoegh-Guldberg et al., 2007) for Heron Island.

Coral nubbins were sampled using sterile forceps at the start of the experiment (t_0_) and after 7 days for both the control (t_final_ Control) and heat stress treatments (t_final_ Heat stress). The nubbins were immediately placed into 15 mL falcon tubes containing 3 mL of RNA*later* (Ambion, Life Technologies, Australia; Vega Thurber et al., 2009), which was a sufficient volume to completely immerse the nubbins. The nubbins were subsequently stored at -80°C until processing.

### Photosynthetic Health of Corals

Photosynthetic health of the corals was checked using a diving pulse amplitude modulated (PAM) fluorometer (Walz, Germany) in the t_final_ Control and t_final_ 31°Ctreatments. Corals were dark-adapted for 10 min before their minimum fluorescence in the dark (F_O_) was recorded. Maximum fluorescence (F_M_) was determined using a saturating pulse of light for 0.8 s. The corals were then illuminated under 616 μmol photon m^-2^ s^-1^ light for 5 min to test their ability to sustain photosynthetic function under light. Maximum Quantum Yield (F_V_/F_M_) was measured on dark-adapted samples and effective quantum yield Y(PSII), regulated non-photochemical quenching Y(NPQ), and non-regulated non-photochemical quenching Y(NO) were measured on light adapted samples. To compare the changes in the F_V_/F_M,_ Y(PSII), Y(NPQ), and Y(NO) measurements in the t_0_, t_final_ Control, and t_final_ Heat Stress treatments, a 1-way analysis of variance (ANOVA) was used (treatment) to determine significant differences (*P* < 0.05) between these measurements. Prior to this, data was tested for normality using the Kolmogorov–Smirnov test and Levene’s test was used for homogeneity of variance.

### Coral-Bacterial Cell Separation

Coral nubbins were thawed slowly on ice and removed from the RNA-later solution using sterile forceps and kimwipes to remove excess solution (Vega Thurber et al., 2009). Replicate nubbins from the same donor colony (A, B, or C) were pooled and placed into sterile 150 mL conical flasks containing 15 mL sterile-autoclaved calcium and magnesium free seawater plus 10 mM EDTA (CMFSWE). The surfaces of the nubbins were airbrushed using 80 psi with a sterile 1 mL barrier tip (fresh tip for each new nubbin) in the conical flasks using sterile forceps to hold the nubbin in place. For each sample, the 15 mL tissue slurry was then filtered through a sterile 100 μm cell strainer (BD 352360) into a sterile 50 mL plastic centrifuge tube to remove host cells. The <100 μm filtrate was then filtered through a 3 μm filter (Whatman) and sterile filter tower apparatus (Nalgene) using vacuum pressure to remove any host cells larger than 3 μm. The resultant <3 μm filtrate (∼15 mL) was centrifuged at 14462 ×*g* to pellet the microbes for 5 min. DNA was extracted from the cell pellet using the MO BIO Ultra Clean Microbial DNA Kit (Carlsbad, CA, USA) according to the manufacturer’s instructions. Genomic DNA concentrations were measured using a Qubit 2.0 fluorometer (Invitrogen).

### 16S rRNA Amplicon Sequencing and Analysis

The bacterial community composition in each nubbin was determined using the universal bacterial 16S rRNA gene primers 27F (5′-AGAGTTTGATCMTGGCTCAG-3′) and 1392R (5′-ACGGGCGGTGTGTRC-3′; resulting in a 1365bp product) and the HotStarTaq Plus Master Mix Kit (Qiagen, USA). A 30 cycle amplification process was employed, incorporating the following cycling conditions: 94°C for 3 min, followed by 28 cycles of 94°C for 30 s, 53°C for 40 s and 72°C for 1 min, after which a final elongation step at 72°C for 5 min was performed. In addition, the composition and diversity of the *Vibrio* community was assessed using the *Vibrio* specific 16S rRNA gene primers VF169 (5′-GGATAACYATTGGAAACGATG-3′; Yong et al., 2006) and Vib2_R (5′-GAAATTCTACCCCCCTACAG-3′; Thompson et al., 2004; Vezzulli et al., 2012), resulting in a 511 bp product. In this instance, Mangomix^TM^ (Bioline) Taq polymerase was used and the following cycling conditions were performed: an initial activation step at 95°C for 120 s, followed by 30 cycles of denaturation at 95°C for 15 s, annealing at 53°C for 30 s and extension at 72°C for 30 s, after which a final elongation step at 72°C for 10 min was performed. In both cases, PCR products were used to prepare DNA libraries with the Illumina TruSeq DNA library preparation protocol. Sequencing was performed, following an additional amplification step using the 27F-519R primer pair for the 16S rRNA amplicon sequences on an Illumina MiSeq (at Molecular Research LP; Shallowater, TX, USA) following the manufacturer’s guidelines.

16S rRNA gene sequences were analyzed using the QIIME pipeline (Caporaso et al., 2010; Kuczynski et al., 2011). De novo Operational Taxonomic Units (OTUs) were defined at 97% sequence identity using UCLUST (Edgar, 2010) and taxonomy was assigned to the Greengenes database (version 13_8; McDonald et al., 2012) using BLAST (Altschul et al., 1990). Chimeric sequences were detected using ChimeraSlayer (Haas et al., 2011) and filtered from the dataset. Sequences were then rarefied to the same depth to remove the effect of sampling effort upon analysis (Santos et al., 2014) and chao1 diversity estimates were calculated. ANOVA was used (treatment) to determine significant differences (*P* < 0.05) between the diversity estimates in each treatment. Prior to this, data was tested for normality using the Kolmogorov–Smirnov test and Levene’s test was used for homogeneity of variance. In cases where these assumptions were not met, log_10_ transformations were performed. The community composition for each of the treatments t_0_, t_final_ Control, and t_final_ Heat Stress was averaged across the three replicates within each treatment.

Multivariate statistical software (PRIMER v6) was used to measure the degree of similarity between the bacterial community composition in each treatment (Clarke and Gorley, 2006). Data was square-root-transformed and the Bray–Curtis similarity was calculated between samples. Similarity percentage (SIMPER) analysis (Clarke, 1993) was used to identify the sequences contributing most to the dissimilarity between the treatments.

For the *Vibrio*-specific assay, the OTUs representing >1% of the total sequences were combined with various *Vibrio* species nucelotides taken from Yong et al. (2006) and *V. coralliilyticus* nucleotides taken from Huete-Stauffer et al. (unpublished), Ben-Haim et al. (2003b) and Ushijima et al. (2014) to build a phylogenetic tree. Sequences were first aligned and inspected using MUSCLE (Edgar, 2004) and the tree was constructed after 1,000 bootstrap re-samplings of the maximum-likelihood method using the Tamura-Nei model (Tamura et al., 2007) in MEGA 6.0 (Tamura et al., 2013), where only values >50% were displayed on the tree (Felsenstein, 1985). The OTU abundance was represented as a percentage of the overall community composition. OTUs were included on the tree if responsible for driving significant differences between the treatments according to SIMPER analysis and were color coded according to whether the OTU was more abundant in the t_final_ Control treatment (blue circle) or the t_final_ Heat stress treatment (red circle).

### Quantitative PCR and Analysis

Quantitative PCR analyses targeting a *Vibrio-* specific region of the 16S rRNA gene and the heat shock protein gene (*dnaJ*) specific to *V. coralliilyticus* were conducted on all samples. Standards were created by growing the bacterial isolates *V. parahaemolyticus* (ATCC 17802) and *V. coralliilyticus* (ATCC BAA-450) overnight in Marine Broth (BD, Difco) at 37°C (150 rpm shaking water bath) and 28°C (170 rpm in a shaking incubator), respectively. Prior to qPCR analysis, calibration curves for each assay were created using viable counts from dilution series of the isolates. The cultures were homogenized and divided into 4 × 1 mL aliquots, washed three times with sterile phosphate-buffered saline (PBS) and pelleted at 5200 g for 10 min. Three of the washed pellets were used for DNA extraction using the MO BIO Ultra Clean Microbial DNA Kit (Carlsbad, CA, USA), while the remaining washed pellet was resuspended in 1 mL PBS and 10-fold serial dilutions with Phosphate Buffered Saline were prepared in triplicate. Three replicate 100 μL aliquots from each dilution (10^-5^–10^-8^) were spread onto marine agar plates and grown at 37°C (*V. parahaemolyticus*) or 28°C (*V. coralliilyticus*) over 24–48 h, and resultant colonies were counted.

A 1:5 dilution of DNA: nuclease free water was used for all qPCR assays to reduce pipetting errors. The *Vibrio* population was assessed using 16S rRNA *Vibrio* primers Vib1_F (5′-GGCGTAAAGCGCATGCAGGT-3′) and Vib2_R (5′-GAAATTCTACCCCCCTACAG-3′; Thompson et al., 2004; Vezzulli et al., 2012) producing a 113 bp product. Power SYBR Select Master Mix (Applied Biosystems) was used, with reaction mixtures comprising 10 μL Master Mix, 5 μL of diluted (1:5) sample, and 0.4 μM of each primer to a final volume of 20 μL. The qPCR was performed using a Step One Plus (Applied Biosystems) and the following optimized cycling conditions: 2 min at 50°C, then an initial denaturation-hot start of 2 min at 95°C, followed by 40 cycles of the two-step reaction: 95°C for 15 s and 60°C for 1 min. This was followed by a holding stage at 72°C for 2 min and a melt curve stage.

The relative abundance of *V. coralliilyticus* was measured by targeting the *dnaJ* gene that encodes heat shock protein 40 in this species (Pollock et al., 2010), using the primers: Vc_dnaJ_F1 (5′-CGGTTCGYGGTGTTTCAAAA-3′) and Vc_dnaJ_R1 (5′-AACCTGACCATGACCGTGACA-3′) and a TaqMan probe, Vc_dna-J_TMP (5′-6-FAM-CAGTGGCGCGAAG-MGBNFQ-3′; 6-FAM; Pollock et al., 2010). Reaction mixtures included a 10 μL TaqMan Universal Master Mix II (Applied Biosystems), 5 μL of diluted (1:5) sample, 0.6 μM of each primer and 0.2 μM fluorophore-labeled TaqMan probe in a final total volume of 20 μL. The optimized qPCR cycling conditions were: 2 min at 50°C, then an initial denaturation-hot start of 10 min at 95°C, followed by 40 cycles of the following incubation pattern: 95°C for 15 s and 60°C for 1 min.

Resultant qPCR data for the *Vibrio*-specific and *V. coralliilyticus* assays were analyzed using Step One Software V2.3 (Applied Biosystems). The concentrations of bacteria were normalized to the coral surface area per cm^2^, which was calculated by paraffin wax dipping as described in Holmes (2008) and Veal et al. (2010). To compare the abundance of bacteria in the t_0_, t_final_ Control, and t_final_ Heat Stress treatments using the qPCR assays, ANOVA was used (treatment) to determine significant differences (*P* < 0.05) between the abundances in each treatment (qPCR). Prior to this, data was tested for normality using the Kolmogorov–Smirnov test and Levene’s test was used for homogeneity of variance. In cases where these assumptions were not met, log_10_ transformations were performed.

## Results

### Effects of Elevated Temperature on Coral Health

No visual signs of stress or bleaching were evident in the Control nubbins over the course of the experiment, yet evidence of bleaching was observed in the Heat Stress nubbins where significant levels of heat stress of the zooxanthellae were detected in the t_final_ Heat Stress treatment compared to the t_final_ Control nubbins using PAM fluorometry. Heat stressed corals showed a strong decline in zooxanthellae condition (significant decrease in the F_V_/F_M_ (*P* = 0.002) and Y[PSII] (*P* = 0.003) measurements; Supplementary Information Tables S1 and S2), while simultaneously the zooxanthellae were protecting their cells from further photodamage by significantly increasing the xanthophyll cycle –Y[NPQ] measurements (Supplementary Information Tables S1 and S2).

### Bacterial Community Composition

**FIGURE 1:**
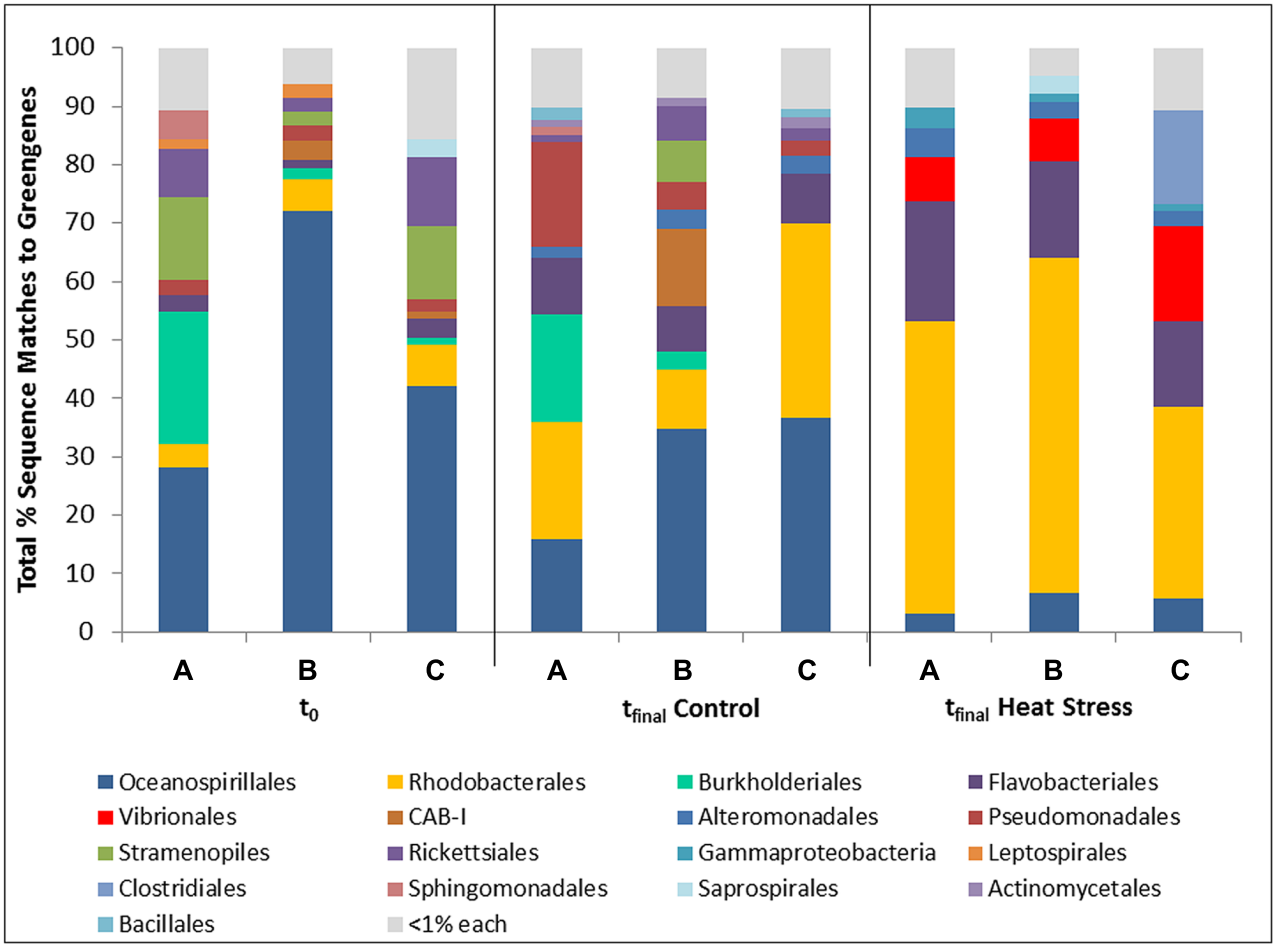
**Bacterial taxa (order) associated with the coral *Pocillopora damicornis* on Heron Island, the Great Barrier Reef at t_0_ (22°C; **A–C** are replicates), t_final_ Control (22°C; **A–C** are replicates), and t_final_ Heat stress (31°C; **A–C are replicate nubbins**) conditions using 16S rRNA gene amplicon sequencing.** Hits were generated by comparing the sequences with BLASTn to the Greengenes database in QIIME.

Differences in bacterial community composition between the t_final_ Control and t_final_ Heat Stressed corals were identified using 16S rRNA gene amplicon sequencing (**Figure 1**). The community composition of the t_final_ Control and t_final_ Heat Stress treatments were 42% dissimilar (SIMPER analysis; Supplementary Information Figure S1, Supplementary Information Table S3), while the largest difference (56%) in the community composition was between t_0_ and the t_final_ Heat Stress treatments (Supplementary Information Table S4). Chao1 diversity estimates revealed that the t_final_ Heat Stress treatment had significantly (*P* < 0.05) higher diversity (1406 ± 155 SD) compared to the t_final_ Control (995 ± 23 SD).

The bacterial community at t_0_ was dominated by the *Oceanospirillales* (47%), which were primarily composed of members from the *Endozoicomonacea*, followed by *Burkholderiales* (8.5%), *Rickettsiales* (7%), and *Rhodobacterales* (6%) (**Figure 1**). A shift in the community was observed in control corals over the 7 day experiment involving an increase in the relative occurrence of sequences matching the *Rhodobacterales* (21%) and *Flavobacteriales* (8.6%), and a decrease in *Oceanospirillales* sequences (29%). These shifts are indicative of a mild experimental effect (**Figure 1**). However, a dramatic community shift was detected in the t_final_ Heat Stress treatment relative to both the t_0_ and t_final_ Control samples, which involved an increase in the relative proportion of *Rhodobacterales* (46.7%), *Flavobacteriales* (17.3%), and *Vibrionales* (10.5%). The occurrence of *Vibrionales* is notable because these organisms were not present in either control treatment (**Figure 1**). SIMPER analysis revealed that the decrease in *Oceanospirillales* abundance and increase in *Vibrionales* abundance were primarily responsible for differences in community composition between the t_final_ Control and Heat Stress treatments (Supplementary Information Table S3).

### Quantification of the General *Vibrio* Population and of *V. coralliilyticus* Using Real Time qPCR

**FIGURE 2:**
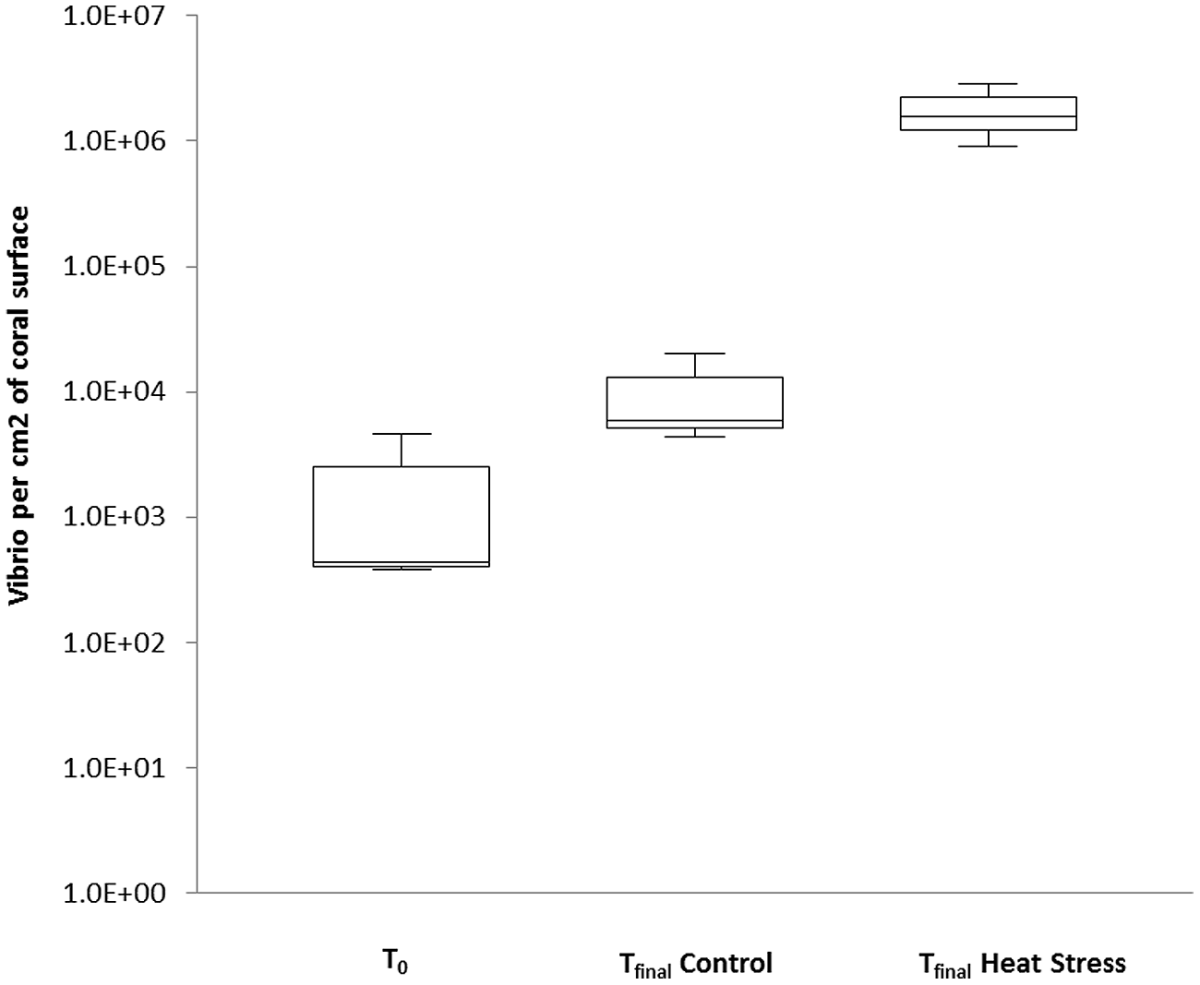
**Quantitative PCR was performed to quantify the abundance of natural populations of Vibrios associated with the coral *P. damicornis* on Heron Island, the Great Barrier Reef at t_0_ (22°C), t_final_ Control (22°C), and t_final_ Heat stress (31°C) conditions.** Standard curve: *R*^2^ = 0.99, Eff% = 93.1. Abundances are expressed as the number of bacteria per cm^2^. *n* = 3.

To confirm and quantify the increased abundance of *Vibrio* observed in t_final_ Heat Stressed corals, we applied a qPCR assay to track changes in the relative abundance of the *Vibrio* community. The *Vibrio* community-specific qPCR assay detected Vibrios in all treatments (**Figure 2**, standard curve: *R*^2^ = 0.99, Efficiency % = 93.1), but abundances were significantly higher in the t_final_ Heat Stress treatment, where they reached an average of 2.2 × 10^7^ (±6.3 × 10^6^ SD) cells cm^-2^ of coral surface (*P* < 0.01, Supplementary Information Table S5). *Vibrio* abundances in this treatment were two–three orders of magnitude higher than in the t_final_ Control [1.4 × 10^5^ (±9.5 × 10^4^ SD) cells cm^-2^] and t_0_ (2.0 × 10^4^ (±1.5 × 10^4^ SD) cells cm^-2^) samples, respectively, (**Figure 2**).

**FIGURE 3:**
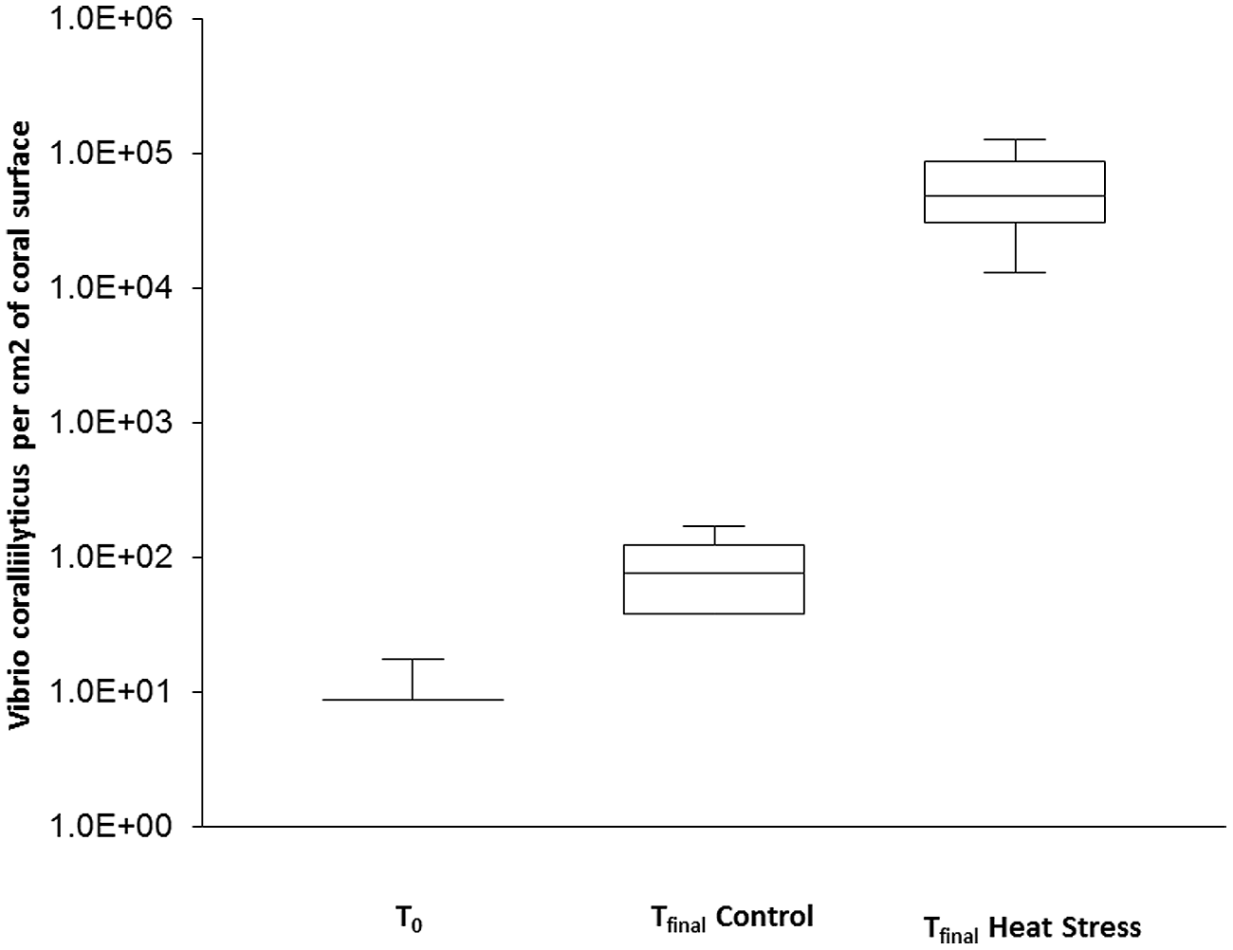
**Quantitative PCR assays were used to quantify the abundance of natural populations of *Vibrio coralliilyticus* associated with the coral *P. damicornis* on Heron Island, the Great Barrier Reef at t_0_ (22°C), t_final_ Control (22°C), and t_final_ Heat stress (31°C) conditions, standard curve: *R*^2^ = 0.995, Eff% = 99.9.** Abundances are expressed as the number of bacteria per cm^2^. *n* = 3.

Variation in the abundance of the coral pathogen *V. coralliilyticus* was also assessed using qPCR. In the t_0_ corals, *V. coralliilyticus* was detected in only one of the three replicate colonies, in very low abundance (17.5 cells cm^-2^ of coral surface). Similarly, low concentrations were observed in the t_final_ Control samples, with abundances in one replicate below the detection limit and a mean of 81.5 cells cm^-2^ observed in the other two replicates. In contrast, *V. coralliilyticus* concentrations in the t_final_ Heat Stress treatment 6.3 × 10^4^ (±3.4 × 10^4^ SD) were significantly higher (*P* < 0.05, Supplementary Information Table S6) and reached up to four orders of magnitude higher than the t_final_ Control (**Figure 3**).

### Characterizing Changes in the *Vibrio* Population Induced by Heat Stress

**FIGURE 4:**
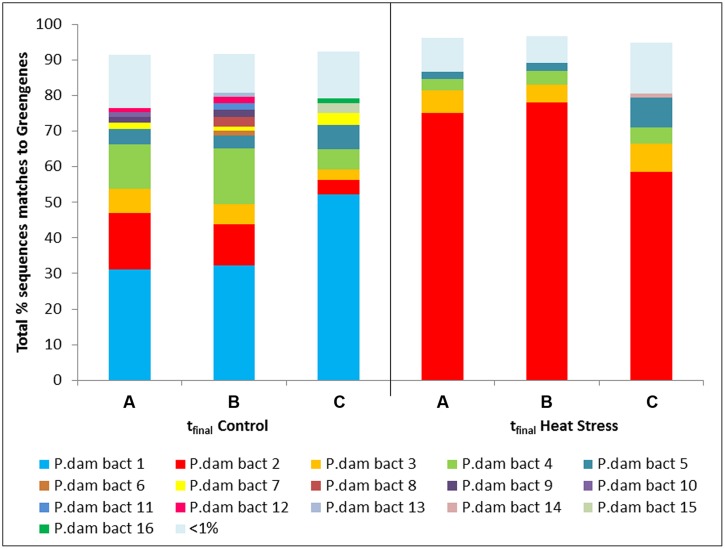
**Operational taxonomic units (OTUs) from the *Vibrio* community associated with the coral *P. damicornis* on Heron Island, the Great Barrier Reef at t_final_ Control (22°C; **A–C are replicate nubbins**) and t_final_ Heat stress (31°C; **A–C are replicate nubbins**) conditions**.

**FIGURE 5:**
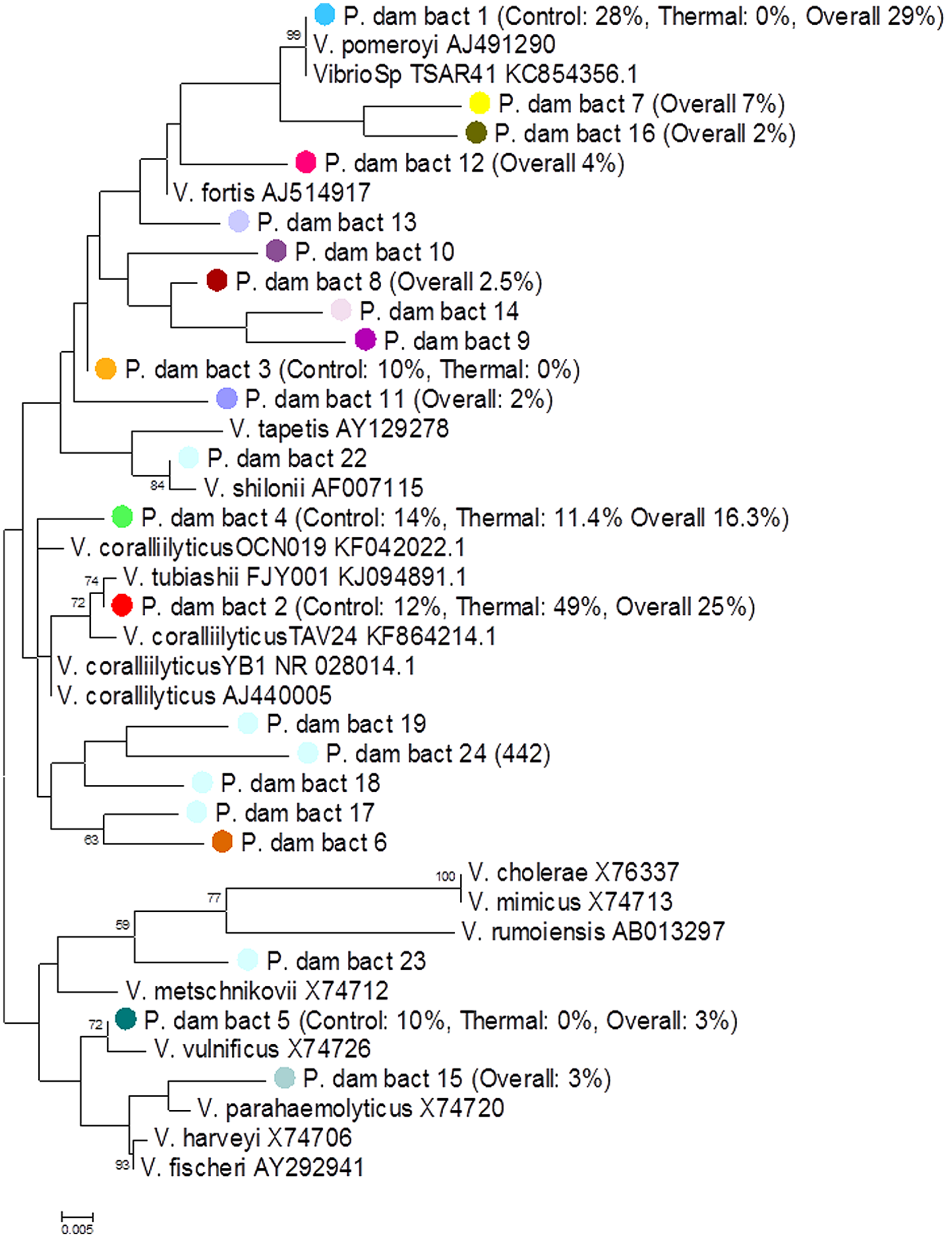
**Phylogenetic tree of the *Vibrio* community associated with the coral *P. damicornis*.** The colors of OTU circles match the color of OTUs from **Figure 4**. The percentage abundances of the OTUs in the t_final_ Control and t_final_ Heat Stress treatments are represented as a percentage of the total community composition only if the OTU is responsible for driving significant differences between the treatments according to SIMPER analysis (Supplementary Table S6). The numbers at the nodes are percentages indicating the levels of bootstrap support, based on 1,000 resampled data sets where only bootstrap values of >50% are shown. The scale bar represents 0.005 substitutions per nucleotide position.

Using a *Vibrio*-specific 16S rRNA amplicon sequencing approach we observed a clear shift in the composition of the coral *Vibrio* community between the t_final_ Control and t_final_ Heat Stress treatments. Consistent with the results of the qPCR assay, where negligible numbers of *Vibrio* were detected, a small number (*n* = 2024) of *Vibrio* sequences were observed in the t_0_ treatment. To avoid rarefying to this very low number of sequences, the t_0_ treatment was subsequently omitted from the data set, as we consider the key comparison to test for the effects of increased seawater temperatures to be the t_final_ Control vs. Heat Stress treatments. The *Vibrio* community composition was different between the t_final_ Control and Heat Stress treatments. In particular, two OTUs, denoted *P. dam* bact 1 and bact 2, were responsible for driving the largest differences (29 and 25%, respectively, according to SIMPER analysis) between treatments (**Figure 4**, Supplementary Information Table S7). While the *P. dam* bact 1 OTU comprised an average of 38.5% (±6.8%) of the community in the t_final_ control treatment (**Figure 4**), it was not present in the t_final_ Heat Stress treatment. In contrast, the *P. dam* bact 2 OTU was more abundant in corals from the t_final_ Heat Stress treatment, comprising an average of 70.6% (±6.0%) of the total *Vibrio* community (**Figure 4**), while representing only 10.4% (±3.4%) of the community in the t_final_ control treatment. Phylogenetic analysis of the two dominant *Vibrio* OTUs (**Figure 5**) revealed that *P. dam* bact 1 appears to be closely related to *V. pomeryoi* (AJ491290), while *P. dam* bact 2 may be related to *V. tubiashii* (KJ094891.1) and *V. coralliilyticus* (KF864214.1; **Figure 5**).

## Discussion

Rising global temperatures, related to anthropogenically driven climate change, are expected to drive the geographical expansion of pathogens and the spread of disease outbreaks (Harvell et al., 1999, 2002; Burge et al., 2014). In marine habitats, a rise in *Vibrio-*induced diseases has been identified as an emerging global issue and has been correlated to rising seawater temperatures (Vezzulli et al., 2012; Baker-Austin et al., 2013). For instance, increasing seawater temperature has been linked to increased *Vibrio* occurrence in the North and Baltic Seas and a concurrent increase in cases of human infections by *Vibrio* species in this region (Vezzulli et al., 2012; Baker-Austin et al., 2013). Similarly, increasing numbers of human infections by *V. vulnificus* and *V. parahaemolyticus* off the coast of Spain have been linked to higher seawater temperatures (Martinez-Urtaza et al., 2010).

Clear links between elevated seawater temperature and the global decline of corals have also become increasingly apparent (Mydlarz et al., 2009; De’ath et al., 2012). Elevated seawater temperatures have led to (i) increased occurrence of coral bleaching, whereby symbiotic dinoflagellates are expelled from the coral host (Hoegh-Guldberg et al., 2007) and (ii) a situation where many corals are living close to their thermal physiological maximum (Hoegh-Guldberg et al., 2007). In addition to these direct effects on coral physiology and the coral-*Symbiodinium* symbiosis, rising seawater temperatures have also been linked to increased incidence of coral disease and microbial-associated bleaching, or white syndrome (Bruno et al., 2007). In particular, *V. shiloi* and *V. coralliilyticus* have been identified as temperature-dependent pathogens responsible for coral bleaching (Kushmaro et al., 1996, 1997, 1998; Ben-Haim and Rosenberg, 2002; Ben-Haim et al., 2003a).

To date, the majority of research investigating the roles of *Vibrio* sp. in coral disease has been conducted in the laboratory using cultured isolates obtained from healthy and diseased corals (Kushmaro et al., 1998; Banin et al., 2000; Ben-Haim and Rosenberg, 2002; Ben-Haim et al., 2003a; Koren and Rosenberg, 2006; Vidal-Dupiol et al., 2011; Garren et al., 2014; Rubio-Portillo et al., 2014) with relatively few studies assessing natural populations of coral-associated *Vibrio* during heat stress or bleaching events (Bourne et al., 2008; Vezzulli et al., 2010). Community finger-printing approaches have previously revealed increases in the relative abundance of *Vibrio* populations during a naturally occurring bleaching event on the GBR (Bourne et al., 2008), while the appearance of *V. coralliilyticus* in diseased specimens of the octocoral *Paramuricea clavata* was also linked to elevated seawater temperature (Vezzulli et al., 2010).

In our study, initial evidence for a temperature induced increase in coral-associated *Vibrio* was provided by 16S rRNA gene amplicon sequencing. Corals from the control treatments were dominated by the *Oceanospirillales*, primarily due to the abundance of *Endozoicomonacea*, a group widely shown to be associated with healthy colonies of diverse coral species (Morrow et al., 2012, 2014; Bayer et al., 2013; Neave et al., 2014) including *P. damicornis* (Bourne and Munn, 2005). In contrast, the bacterial community in t_final_ Heat Stressed corals was characterized by significantly higher levels of diversity (Chao1) than the t_final_ Control corals. This is consistent with previous studies where diversity increased among white plague affected corals (Sunagawa et al., 2009). The t_final_ Heat Stressed corals contained diverse assemblages of copiotrophic and potentially opportunistic microbes including *Rhodobacteriales*, *Flavobacteriales*, and *Vibrionales*. Notably, while *Vibrio* sequences made up 10.5% of sequences in corals from the t_final_ Heat Stress treatment, they were completely absent in the t_0_ and t_final_ Control samples. In addition, a substantial decrease in *Oceanospirillales* and a disappearance of *Burkholderiales* was observed in t_final_ Heat Stressed corals. The changes observed here are consistent with previous research indicating that specific bacterial populations, including putative pathogens, emerge, and dominate the coral-associated bacterial community during environmental stress events (Roder et al., 2014). These community shifts may be a direct effect of temperature on the growth of specific members of the microbial community, or alternatively caused by a change in the chemicals released by heat-stressed corals (Garren et al., 2014).

Due to the increased proportion of *Vibrio* sequences in the 16S rRNA amplicon analysis and the potential role of *Vibrio* in coral disease (Vezzulli et al., 2010), we investigated the dynamics of this community further using targeted qPCR and *Vibrio*-specific amplicon sequencing approaches. A clear shift in the composition of the *Vibrio* community was observed in conjunction with the increased *Vibrio* abundance under elevated seawater temperature. Using qPCR, we detected low abundances of total *Vibrio* in the t_0_ and t_final_ Control treatments, consistent with previous observations in healthy corals (Ritchie and Smith, 2004; Raina et al., 2009; Vezzulli et al., 2013) and our 16S rRNA amplicon sequencing results. However, we observed an increase in relative *Vibrio* abundance of two–three orders of magnitude in the t_final_ Heat Stressed corals. These patterns support previous reports that *Vibrio* abundance is linked to seawater temperature (Rubio-Portillo et al., 2014). While the increased abundance of *V. coralliilyticus* is part of a broader increase in abundance of total Vibrios, the magnitude of increase was substantially higher in *V. coralliilyticus* (four orders of magnitude compared to 2–3 orders of magnitude). This indicates that the putative coral pathogen *V. coralliilyticus* particularly benefited from the increased seawater temperature during in this study.

The ecological role of the resident *Vibrio* community in the health of corals is likely to vary substantially across species. Some Vibrios appear to form mutualistic relationships with corals by fixing nitrogen in the mucus (Chimetto et al., 2008) whereas others are putative agents of coral disease. However, despite substantial evidence of links between coral disease and *Vibrio* occurrence, in many cases it is unknown whether these organisms are the primary etiological agents or simply opportunistic colonizers that exploit the coral when host health is compromised (Bourne et al., 2008; Raina et al., 2010). While difficulties in assigning *Vibrio* taxonomy using 16S rRNA sequencing approaches are sometimes encountered (Cana-Gomez et al., 2011), our *Vibrio* specific 16S amplicon assay demonstrated clear differences between the *Vibrio* communities in the control and heat-stress samples and identified two key OTUs responsible for driving these differences. In the control corals the *Vibrio* community was dominated by OTUs that matched *V. pomeroyi* (AJ491290), supporting previous research showing *V. pomeryoi* is found year round in healthy corals (Rubio-Portillo et al., 2014). *V. pomeryoi* is not known to be involved in coral disease and is likely a normal resident member of the coral-associated community (Rubio-Portillo et al., 2014). Up to 70% of the *Vibrio* community in t_final_ Heat Stressed corals was comprized of a single OTU (OTU *P. dam* bact 2), which our phylogenetic analysis indicates is closely related to the oyster pathogen *V. tubiashii* (KJ094891.1; Hada et al., 1984; Hasegawa et al., 2008; Richards et al., 2015) and the coral pathogen *V. coralliilyticus* (KF864214.1). *V. tubiashii* and *V. coralliilyticus* are highly related species (Ben-Haim et al., 2003b), and whilst the taxonomy of OTU *P. dam* bact 2 remains to be fully resolved, the phylogenetic positioning close to several *V. coralliilyticus* strains indicates that this organism may be *V. coralliilyticus*. This would be consistent with the findings of our *V. coralliilyticus* qPCR analysis, where a four orders of magnitude increase in abundance of *V. coralliilyticus* was observed in corals from the t_final_ Heat Stress treatment. These results are consistent with findings of Vezzulli et al. (2010) who only observed *V. coralliilyticus* in diseased coral specimens, as well as Ben-Haim and Rosenberg (2002) who, using cultured isolates of *V. coralliilyticus*, demonstrated that elevated temperatures are crucial to the infection of *P. damicornis*.

Our findings demonstrate, for the first time, that elevated seawater temperature increases the abundance and alters the composition of an environmental *Vibrio* community occurring among a mixed natural microbial community associated with the ecologically important coral species *P. damicornis*. Importantly, these microbial shifts involve a dramatic rise in the relative abundance of pathogens including *V. coralliilyticus.* Our research builds upon previous studies using cultured isolates, to highlight that natural populations of Vibrios, occurring within mixed natural communities of coral associated microbes may rise to prominence under heat stress conditions. Currently, up to a third of all coral species face extinction (Carpenter et al., 2008), with coral disease recognized as a significant and increasing threat. Our data provide direct quantitative support for the theory that increasing sea surface temperature occurring as a result of climate change, will affect coral reefs by promoting an increase in the abundance of coral pathogens.

## Conflict of Interest Statement

The authors declare that the research was conducted in the absence of any commercial or financial relationships that could be construed as a potential conflict of interest.
