# Supplementary material for: Increased seawater temperature increases the abundance and alters the structure of natural Vibrio populations associated with the coral Pocillopora damicornis
Source: Front Microbiol. 2015 May 18;6:432. doi: 10.3389/fmicb.2015.00432 (PMC4435422; doi:10.3389/fmicb.2015.00432)
Supplement: Supplementary file 1 [file Data_Sheet_1.PDF]

frontiers

Supplementary Material

Increased seawater temperature increases the abundance and alters the structure of natural populations of *Vibrio* associated with the coral *Pocillopora damicornis*

Jessica Tout[1*], Nachshon Siboni[1], Lauren F. Messer[1], Melissa Garren[2], Roman Stocker[2], Nicole S. Webster[3], Peter J. Ralph[1], Justin R. Seymour[1]

[1]Plant Functional Biology & Climate Change Cluster, University of Technology, Sydney, NSW, Australia.

[2] Ralph M. Parsons Laboratory, Department of Civil and Environmental Engineering, Massachusetts Institute of Technology, Cambridge MA USA.

[3]Australian Institute of Marine Science, Townsville, QLD, Australia.

Correspondence:
Jessica Tout
Plant Functional Biology & Climate Change Cluster,
PO Box 123 Broadway
University of Technology Sydney, 2007, NSW, Australia.
Jessica.Tout@uts.edu.au

Table 1 Photosynthetic health of the corals were measured using PAM fluorometry was used at t_{final} Control and t_{final} Heat Stress treatments.

Measurement/ Treatment	Maximum quantum yield of PSII (F_V/F_M)	Effective quantum yield of Y(PSII)	Nonregulated nonphotochemical quenching (Y(NO))	Regulated non photochemical quenching (YNPQ)
**t_{final} Control**	64.8%	42.7%	23.6%	33.6%
**t_{final} Heat Stress**	51%	21.4%	28.9%	49.6%

Table 2: A 1-way ANOVA was used to determine significant differences between the PAM fluorometry measurements at each treatment.

ANOVA

		Sum of Squares	df	Mean Square	F	Sig.
**F_VF_M**	Between Groups	288.592	1	288.592	45.705	.002 ***
	Within Groups	25.257	4	6.314		
	Total	313.849	5			
**Y(PSII)**	Between Groups	680.328	1	680.328	40.757	.003 ***
	Within Groups	66.770	4	16.692		
	Total	747.098	5			
**Y(NO)**	Between Groups	41.910	1	41.910	.867	.405
	Within Groups	193.434	4	48.358		
	Total	235.344	5			
**Y(NPQ)**	Between Groups	384.521	1	384.521	18.271	.013 ***
	Within Groups	84.182	4	21.045		
	Total	468.703	5			

Table 3: PRIMER table showing SIMPER analysis between the t_{final} Control and t_{final} Heat Stress treatments with an average dissimilarity of 41.78%.

Groups Thermal & control
Average dissimilarity = 41.78

Species	Thermal Av. Abund	Control Av. Abund	Av. Diss	Diss /SD	Contrib %	Cum .%
k__Bacteria;p__Proteobacteria;c__Gammaproteobacteria;o__Oceanospirillales	0.22	0.53	3.92	2.79	9.39	9.39
k__Bacteria;p__Proteobacteria;c__Gammaproteobacteria;o__Vibrionales	0.32	0.06	3.22	4.32	7.7	17.09
k__Bacteria;p__Proteobacteria;c__Alphaproteobacteria;o__Rhodobacterales	0.68	0.45	2.98	1.66	7.14	24.23
k__Bacteria;p__Proteobacteria;c__Gammaproteobacteria;o__Pseudomonadales	0.08	0.27	2.39	1.61	5.72	29.95
k__Bacteria;p__Proteobacteria;c__Betaproteobacteria;o__Burkholderiales	0.07	0.23	2.08	1.1	4.99	34.94
k__Bacteria;p__Cyanobacteria;c__Chloroplast;o__CAB-I	0.03	0.17	1.71	0.91	4.08	39.02
k__Bacteria;p__Firmicutes;c__Clostridia;o__Clostridiales	0.14	0.02	1.59	0.71	3.8	42.82
k__Bacteria;p__Cyanobacteria;c__Chloroplast;o__Stramenopiles	0.02	0.15	1.58	1.36	3.79	46.61
k__Bacteria;p__Bacteroidetes;c__Flavobacteriia;o__Flavobacteriales	0.41	0.29	1.55	3.39	3.7	50.31
k__Bacteria;p__Proteobacteria;c__Alphaproteobacteria;o__Rickettsiales	0.07	0.16	1.17	1.59	2.79	53.1
k__Bacteria;p__Actinobacteria;c__Actinobacteria;o__Actinomycetales	0.05	0.12	0.99	3.29	2.37	55.48
k__Bacteria;p__Proteobacteria;c__Alphaproteobacteria;o__RF32	0.01	0.08	0.94	2.51	2.25	57.72
k__Bacteria;p__Bacteroidetes;c__[Saprospirae];o__[Saprospirales]	0.1	0.04	0.81	1.04	1.93	59.66
k__Bacteria;p__Firmicutes;c__Bacilli;o__Bacillales	0.05	0.11	0.8	1.54	1.92	61.58
k__Bacteria;p__Proteobacteria;c__Gammaproteobacteria;o__	0.14	0.08	0.78	1.51	1.87	63.45
k__Bacteria;p__Proteobacteria;c__Alphaproteobacteria;o__BD7-3	0.09	0.04	0.66	2.1	1.59	65.04
k__Bacteria;p__Proteobacteria;c__Alphaproteobacteria;o__Sphingomonadales	0.03	0.09	0.66	1.7	1.58	66.61
k__Bacteria;p__GN02;c__BD1-5;o__	0.03	0.08	0.64	1.34	1.53	68.14

k__Bacteria;p__Firmicutes;c__Bacilli; o__Lactobacillales	0.02	0.07	0.62	1.86	1.49	69.64
k__Bacteria;p__Proteobacteria;c__Deltaproteobacteria;o__Bdellovibrionales	0.09	0.05	0.52	1.43	1.24	70.87
k__Bacteria;p__Proteobacteria;c__Deltaproteobacteria;o__Myxococcales	0.09	0.05	0.5	0.92	1.19	72.06
k__Bacteria;p__Proteobacteria;c__Alphaproteobacteria;o__Rhizobiales	0.1	0.09	0.47	1.44	1.13	73.19
k__Bacteria;p__Bacteroidetes;c__Sphingobacteriia;o__Sphingobacteriales	0	0.04	0.45	0.85	1.08	74.27
k__Bacteria;p__Bacteroidetes;c__Cytophagia;o__Cytophagales	0.06	0.04	0.44	1.43	1.05	75.32
k__Bacteria;p__Proteobacteria;c__Gammaproteobacteria;o__Alteromonadales	0.18	0.17	0.43	1.42	1.03	76.35
k__Bacteria;p__Proteobacteria;c__Epsilonproteobacteria;o__Campylobacterales	0.03	0.02	0.43	1.03	1.03	77.37
k__Bacteria;p__Cyanobacteria;c__ML635J-21;o__	0.03	0	0.41	7.11	0.97	78.34
k__Bacteria;p__Proteobacteria;c__Deltaproteobacteria;o__GMD14H09	0.04	0	0.4	3.24	0.96	79.3
k__Bacteria;p__Fibrobacteres;c__Fibrobacteria;o__Fibrobacterales	0	0.03	0.35	0.67	0.83	80.13
k__Bacteria;p__Proteobacteria;c__Alphaproteobacteria;o__Kordiimonadales	0.03	0	0.34	1.31	0.82	80.94
k__Bacteria;p__Proteobacteria;c__Gammaproteobacteria;o__Legionellales	0.03	0.06	0.34	2.73	0.81	81.76
k__Bacteria;p__Proteobacteria;c__Betaproteobacteria;o__Rhodocyclales	0.01	0.03	0.34	1.1	0.81	82.57
No blast hit;Other;Other;Other	0.01	0.03	0.3	1.87	0.73	83.3
k__Bacteria;p__Planctomycetes;c__BD7-11;o__	0	0.02	0.29	0.67	0.7	84
k__Bacteria;p__Bacteroidetes;c__Bacteroidia;o__Bacteroidales	0.03	0.05	0.29	1.2	0.7	84.7
k__Bacteria;p__Proteobacteria;c__Gammaproteobacteria;o__Xanthomonadales	0	0.02	0.26	2.05	0.62	85.32
k__Bacteria;p__Proteobacteria;c__Gammaproteobacteria;o__[Marinicellales]	0.02	0.03	0.26	1.34	0.61	85.94
k__Bacteria;p__Proteobacteria;c__Gammaproteobacteria;o__Thiohalorhabdales	0.01	0.02	0.25	1.18	0.6	86.53
k__Bacteria;p__Cyanobacteria;c__Synechococcophycideae;o__Synechococcales	0.01	0.02	0.25	0.93	0.59	87.12
k__Bacteria;p__Cyanobacteria;c__Synechococcophycideae;o__Pseudanabae	0	0.02	0.25	1.25	0.59	87.71

nales						
k__Bacteria;p__Cyanobacteria;c__Chloroplast;o__Streptophyta	0.01	0.02	0.24	0.94	0.56	88.27
k__Bacteria;p__Planctomycetes;c__Planctomycetia;o__Pirellulales	0.01	0.02	0.23	0.93	0.55	88.82
k__Bacteria;p__Cyanobacteria;c__Chloroplast;o__Cryptophyta	0	0.02	0.23	0.67	0.55	89.37
k__Bacteria;p__Proteobacteria;c__Gammaproteobacteria;o__HTCC2188	0.01	0.01	0.21	1.39	0.49	89.86
k__Bacteria;p__Proteobacteria;c__Alphaproteobacteria;o__Caulobacterales	0.01	0.02	0.2	1.08	0.48	90.35

Table 4: PRIMER table showing SIMPER analysis between the t_{final} Heat Stress and t_0 treatments with an average dissimilarity of 56.02%.

Groups Thermal & t0
Average dissimilarity = 56.02

Species	Thermal Av. Abund	t0 Av. Abund	Av. Diss	Diss/SD	Contrib%	Cum.%
k__Bacteria;p__Proteobacteria;c__Gammaproteobacteria;o__Oceanospirillales	0.22	0.68	5.73	2.5	10.23	10.23
k__Bacteria;p__Proteobacteria;c__Alphaproteobacteria;o__Rhodobacterales	0.68	0.23	5.59	3.91	9.99	20.22
k__Bacteria;p__Proteobacteria;c__Gammaproteobacteria;o__Vibrionales	0.32	0.03	3.5	4.46	6.24	26.46
k__Bacteria;p__Cyanobacteria;c__Chloroplast;o__Stramenopiles	0.02	0.29	3.32	2.93	5.92	32.39
k__Bacteria;p__Bacteroidetes;c__Flavobacteriia;o__Flavobacteriales	0.41	0.16	3.21	4.23	5.72	38.11
k__Bacteria;p__Proteobacteria;c__Alphaproteobacteria;o__Rickettsiales	0.07	0.26	2.27	2.37	4.05	42.16
k__Bacteria;p__Proteobacteria;c__Betaproteobacteria;o__Burkholderiales	0.07	0.24	2.08	0.97	3.71	45.87
k__Bacteria;p__Firmicutes;c__Clostridia;o__Clostridiales	0.14	0.02	1.56	0.7	2.79	48.67
k__Bacteria;p__Proteobacteria;c__Gammaproteobacteria;o__Alteromonadales	0.18	0.06	1.51	3.17	2.69	51.35
k__Bacteria;p__Spirochaetes;c__[Leptospirae];o__[Leptospirales]	0	0.1	1.26	1.34	2.25	53.61
k__Bacteria;p__Proteobacteria;c__Gammaproteobacteria;o__	0.14	0.04	1.2	2.57	2.14	55.74
k__Bacteria;p__Cyanobacteria;c__Chloroplast;o__CAB-I	0.03	0.12	1.13	1.45	2.02	57.76
k__Bacteria;p__Planctomycetes;c__Planctomycetia;o__Pirellulales	0.01	0.09	1.08	1.96	1.92	59.68
k__Bacteria;p__Proteobacteria;c__Gammaproteobacteria;o__Pseudomonadales	0.08	0.16	0.98	4.1	1.75	61.43
k__Bacteria;p__Proteobacteria;c__Alphaproteobacteria;o__Sphingomonadales	0.03	0.1	0.9	0.86	1.61	63.04
k__Bacteria;p__Bacteroidetes;c__[Saprospirae];o__[Saprospirales]	0.1	0.09	0.84	1.06	1.49	64.53

k__Bacteria;p__Proteobacteria;c__Deltaproteobacteria;o__Bdellovibrionales	0.09	0.03	0.73	1.52	1.31	65.84
k__Bacteria;p__Proteobacteria;c__Alphaproteobacteria;o__BD7-3	0.09	0.03	0.73	4.18	1.3	67.14
k__Bacteria;p__Proteobacteria;c__Deltaproteobacteria;o__Myxococcales	0.09	0.04	0.67	1.13	1.2	68.34
k__Bacteria;p__Acidobacteria;c__Sva0725;o__Sva0725	0	0.06	0.67	0.94	1.2	69.53
k__Bacteria;p__Planctomycetes;c__Planctomycetia;o__Planctomycetales	0	0.05	0.6	4.4	1.07	70.6
k__Bacteria;p__Proteobacteria;c__Gammaproteobacteria;o__[Marinicellales]	0.02	0.06	0.53	1.12	0.94	71.54
k__Bacteria;p__GN02;c__BD1-5;o__	0.03	0.05	0.47	1.71	0.84	72.39
k__Bacteria;p__Fibrobacteres;c__Fibrobacteria;o__Fibrobacterales	0	0.04	0.46	1.41	0.82	73.21
k__Bacteria;p__Proteobacteria;c__Alphaproteobacteria;o__Rhizobiales	0.1	0.08	0.45	1.26	0.8	74.01
k__Bacteria;p__Proteobacteria;c__Epsilonproteobacteria;o__Campylobacterales	0.03	0.02	0.44	0.99	0.78	74.79
k__Bacteria;p__Bacteroidetes;c__Sphingobacteriia;o__Sphingobacteriales	0	0.04	0.44	1.18	0.78	75.57
No blast hit;Other;Other;Other	0.01	0.04	0.44	3.45	0.78	76.35
k__Bacteria;p__Cyanobacteria;c__Oscillatoriophycideae;o__Chroococcales	0	0.03	0.42	5.92	0.75	77.1
k__Bacteria;p__Bacteroidetes;c__Cytophagia;o__Cytophagales	0.06	0.06	0.41	1.25	0.74	77.84
k__Bacteria;p__Bacteroidetes;c__Bacteroidia;o__Bacteroidales	0.03	0.05	0.41	1.07	0.73	78.58
k__Bacteria;p__Spirochaetes;c__[Brachyspirae];o__[Brachyspirales]	0	0.03	0.4	1.33	0.72	79.29
k__Bacteria;p__Verrucomicrobia;c__Verrucomicrobiae;o__Verrucomicrobiales	0	0.03	0.37	1.93	0.66	79.95
k__Bacteria;p__Cyanobacteria;c__ML635J-21;o__	0.03	0	0.37	2.98	0.65	80.6
k__Bacteria;p__Proteobacteria;c__Alphaproteobacteria;o__Caulobacterales	0.01	0.03	0.33	0.87	0.58	81.19
k__Bacteria;p__Proteobacteria;c__Deltaproteobacteria;o__Spirobacillales	0	0.03	0.32	1.53	0.57	81.75
k__Bacteria;p__Proteobacteria;c__Alphaproteobacteria;o__Kordiimonadales	0.03	0	0.3	1.19	0.54	82.29
k__Bacteria;p__Actinobacteria;c__Actinobacteria;o__Actinomycetales	0.05	0.06	0.29	1.41	0.52	82.81
k__Bacteria;p__Proteobacteria;c__Gammaproteobacteria;o__Chromatiales	0	0.03	0.29	3.05	0.51	83.32
k__Bacteria;p__Proteobacteria;c__Alp	0.02	0.04	0.29	1.22	0.51	83.83

haproteobacteria;o__Rhodospirillales						
k__Bacteria;p__Cyanobacteria;c__Chloroplast;o__Haptophyceae	0	0.02	0.28	3.11	0.51	84.34
k__Bacteria;p__Actinobacteria;c__Acidimicrobiia;o__Acidimicrobiales	0	0.03	0.28	1.46	0.5	84.84
k__Bacteria;p__GN04;c__MSB-5A5;o__	0	0.02	0.27	0.67	0.48	85.32
k__Bacteria;p__Proteobacteria;c__Deltaproteobacteria;o__GMD14H09	0.04	0.01	0.27	1.52	0.48	85.81
k__Bacteria;p__Proteobacteria;c__Alphaproteobacteria;o__Kiloniellales	0.01	0.03	0.27	1.13	0.48	86.29
k__Bacteria;p__Planctomycetes;c__028H05-P-BN-P5;o__	0	0.02	0.26	1.09	0.47	86.76
k__Bacteria;p__Cyanobacteria;c__Synechococcophycideae;o__Pseudanabaenales	0	0.02	0.26	1.3	0.47	87.23
k__Bacteria;p__GN02;c__;o__	0	0.02	0.25	0.67	0.44	87.67
k__Bacteria;p__Firmicutes;c__Bacilli;o__Bacillales	0.05	0.04	0.24	1.27	0.42	88.1
k__Bacteria;p__Proteobacteria;c__Betaproteobacteria;o__Tremblayales	0	0.02	0.23	0.78	0.42	88.51
k__Bacteria;p__Proteobacteria;c__Deltaproteobacteria;o__MIZ46	0	0.02	0.23	0.83	0.41	88.92
k__Bacteria;p__Proteobacteria;c__Gammaproteobacteria;o__Thiohalorhabdales	0.01	0.01	0.21	1.25	0.38	89.3
k__Bacteria;p__WPS-2;c__;o__	0	0.02	0.21	1.28	0.37	89.68
k__Bacteria;p__TM6;c__SJA-4;o__	0	0.01	0.19	0.8	0.34	90.02

Table 5: A 1-way ANOVA was used to determine the significant differences between the abundances of Vibrios per cm^2 of *P. damicornis* for each treatment

One-way ANOVA: Abundance of Vibrio per cm^2 of P. damicornis V treatment

Source	DF	SS	MS	F	P
**SAMPLE**	2	9.76072E+14	4.88036E+14	12.40	0.007
**Error**	6	2.36120E+14	3.93533E+13		
**Total**		8	1.21219E+15		

S = 6273223 R-Sq = 80.52% R-Sq(adj) = 74.03%

Table 6: A 1-way ANOVA was used to determine the significant differences between the abundances of *V. coralliilyticus* per cm^2 of *P. damicornis* for each treatment

SOURCE	DF	SS	MS	F	P
A: C1	2	102350	51174.98	10.25	0.011615*
S(A)	6	29965.56	4994.261		
Total (Adjusted)	8	132315.5			
Total	9				

* Term significant at alpha = 0.05

Table 7: PRIMER table showing SIMPER analysis between the OTUs from the t_{final} Heat Stress and t_{final} Control treatments with an average dissimilarity of 46.43%.

CONTROL & THERMAL
Average dissimilarity = 46.43

	**CONTROL**	**THERMAL**				
**Species**	**Av.Abund**	**Av.Abund**	**Av.Diss**	**Diss/SD**	**Contrib%**	**Cum.%**
P.dam bacteria OTU 1	6.16	0	13.75	5.57	29.62	29.62
P.dam bacteria OTU 2	3.13	8.39	11.77	4.05	25.35	54.97
P.dam bacteria OTU 7	1.43	0	3.21	3.48	6.91	61.87
P.dam bacteria OTU 4	3.28	1.98	2.85	1.9	6.13	68.01
P.dam bacteria OTU 9	0.91	0	1.95	1.33	4.21	72.21
P.dam bacteria OTU 12	0.8	0	1.72	1.32	3.71	75.92
P.dam bacteria OTU 5	2.21	1.94	1.64	2.3	3.52	79.44
P.dam bacteria OTU 15	0.56	0	1.31	0.67	2.82	82.26
<1%	3.6	3.2	1.24	1.47	2.66	84.92
P.dam bacteria OTU 8	0.55	0	1.15	0.67	2.48	87.4
P.dam bacteria OTU 11	0.45	0	0.94	0.67	2.03	89.43
P.dam bacteria OTU 16	0.4	0	0.93	0.67	2	91.43

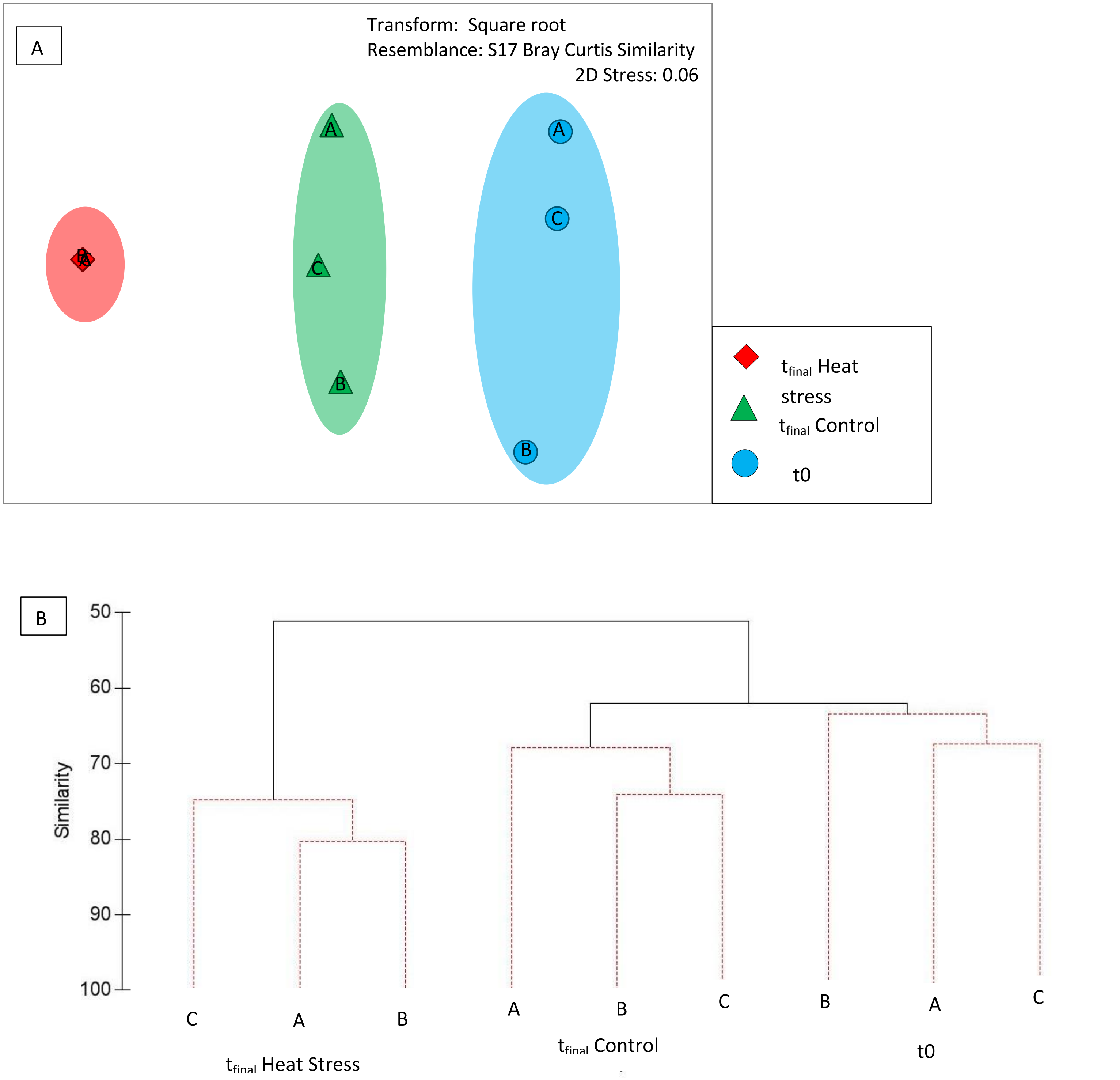

Fig. 1 a) nMDS plot and **b)** CLUSTER analysis of the three treatments t_0, t_{final} Control and t_{final} Heat Stress.
